# Paclitaxel-activated astrocytes produce mechanical allodynia in mice by releasing tumor necrosis factor-α and stromal-derived cell factor 1

**DOI:** 10.1186/s12974-019-1619-9

**Published:** 2019-11-10

**Authors:** Xiaojuan Liu, Raquel Tonello, Yuejuan Ling, Yong-Jing Gao, Temugin Berta

**Affiliations:** 1 Department of Anesthesiology, Pain Research Center, University of Cincinnati College of Medicine, Cincinnati, OH USA; 2 Department of Pathogen Biology, Medical College, Nantong University, Nantong, Jiangsu China; 3 Institute of Pain Medicine, Nantong University, Nantong, Jiangsu China; 4 Institute of Special Environmental Medicine, Nantong University, Nantong, Jiangsu China

**Keywords:** Paclitaxel-associated acute pain syndrome, Paclitaxel, Neuroinflammation, Astrocytes, Tumor necrosis factor, Stromal-derived cell factor

## Abstract

**Background:**

Paclitaxel is a widely used and potent chemotherapeutic agent for the treatment of cancer. However, patients receiving paclitaxel often develop an acute pain syndrome for which there are few treatment options. Astrocytes play an important role in the pathogenesis of pain in multiple preclinical models, as well as in paclitaxel-treated rodents. However, it is still unclear what the exact contribution of astrocytes may be in paclitaxel-associated acute pain syndrome (P-APS).

**Methods:**

P-APS was modeled by a single systemic or intrathecal injection of paclitaxel and astrocyte contribution tested by immunohistochemical, pharmacological, and behavioral approaches. Cell cultures were also prepared to assess whether paclitaxel treatment directly activates astrocytes and whether intrathecal injection of paclitaxel-treated astrocytes produces pain that is reminiscent of P-APS.

**Results:**

Systemic injection of paclitaxel resulted in increased expression of glial fibrillary acidic protein (a common marker of astrocytic activation), as well as both systemic or intrathecal injection of paclitaxel induced pain hypersensitivity indicated by the development of mechanical allodynia, which was significantly reversed by the astrocytic inhibitor L-α-AA. Cultured astrocytes were activated by paclitaxel with significant increases in protein levels for tumor necrosis factor-α (TNF-α) and stromal-derived cell factor 1 (SDF-1). Importantly, intrathecal injection of paclitaxel-activated astrocytes produced mechanical allodynia that was reversed by TNF-α and SDF-1 neutralizing antibodies.

**Conclusion:**

Our results suggest for the first time that paclitaxel can directly activate astrocytes, which are sufficient to produce acute pain by releasing TNF-α and SDF-1. Targeting astrocytes and these cytokines may offer new treatments for P-APS.

## Background

The American Cancer Society estimated that 1.7 million people would be diagnosed with cancer in 2018 and that more than 15.5 million people are living with a history of cancer in the USA today. Paclitaxel (i.e., Taxol and Onxal) is one of the most effective chemotherapeutic drugs, widely used for the treatment of solid cancers such as ovarian, breast, and lung carcinoma [1]. However, paclitaxel is associated with a peculiar syndrome of acute pain, which is described in up to 70% of patients and usually develops within 1 to 3 days of its administration [2, 3]. Although the paclitaxel-associated acute pain syndrome (P-APS) is distinguished by nature and temporal profile from paclitaxel-induced peripheral neuropathy [4], a potential relationship may exist between these two entities and drug doses [2, 4, 5]. It has been reported that patients with higher and uncontrolled P-APS are at higher risk to develop chronic neuropathy [2]. Unfortunately, current therapeutic approaches for P-APS and peripheral neuropathy are limited, and often dose reduction or termination of the treatment are the only options, potentially affecting the optimal care of these patients [2, 6]. Identification of target-directed therapeutic approaches based on mechanistic insights is of paramount significance to improve the treatment of cancer patients [7, 8].

Peripheral neuropathological changes in response to chemotherapeutic drugs are considered the provocative processes for the development of P-APS and peripheral neuropathy [9–11]. However, paclitaxel pain emerges long before deficits in peripheral sensory nerve function and at a dose that causes any nerve deficits [1, 12, 13]. Accumulating evidence is emerging of important roles for glial cells (i.e., satellite glial cells, microglia, and astrocytes) in these syndromes [14–17]. In particular, there is strong evidence for early activation and contribution of astrocytes to peripheral neuropathy [18–20]. Concordantly, pharmacological blockade of astrocytes with general inhibitors of glial cells has been shown to significantly attenuate chemotherapy-induced neuropathic pain in rodents [18, 20, 21], but the fine molecular mechanisms underlying their activation and contribution to P-APS are still incompletely known.

Systemic injection of paclitaxel induces a rapid and persistent activation of spinal astrocytes assessed by increase of glial fibrillary acidic protein (GFAP), a common marker for astrocyte activation [14], and downregulation of astrocytic glutamate transporters [18, 20]. Furthermore, paclitaxel induces robust increases of the phosphorylation of c-jun N-terminal kinase (p-JNK) [20], a member of the mitogen-activated protein kinase family that is associated with astrocytic cytokine production, enhancing the hyperactivity in the spinal pain circuits and promoting neuropathic pain [22, 23].

To date, it is unclear how paclitaxel directly activates astrocytes and whether this activation is sufficient to induce pain. Given that astrocytic end-feet ensheath the microvasculature, small amounts of paclitaxel can cross the blood-brain barrier, and paclitaxel was detected in cerebral fluid [24, 25], we hypothesize that small amounts of paclitaxel are sufficient to directly activate astrocytes and thus elicit P-APS. Here, we show for the first time that small amounts of spinal paclitaxel are sufficient to induce pain hypersensitivity via astrocytes and that paclitaxel directly elicits astrocyte activation and production cytokines that may underlie the paclitaxel-associated acute pain syndrome.

## Materials and methods

### Animals

Wild-type CD1 mice (115 male and 20 female mice, 8–10 weeks old for in vivo studies and P1–3 for in vitro studies) were purchased from Charles River and used as indicated for behavioral, cell culture, and biochemical experiments. Mice were housed four per cage at 22 ± 0.5 °C under a controlled 14/10 h light/dark cycle with free access to food and water. All animal procedures were approved by the University of Cincinnati Institutional Animal Care and Use Committee, and all efforts were made to minimize animal suffering, reduce the number of animals used, and use alternatives to in vivo techniques, in accordance with the International Association for the Study of Pain, the National Institutes of Health Office of Laboratory Animal Welfare Guide for the Care and Use of Laboratory Animals.

### Chemotherapeutic treatment

Paclitaxel (6 mg/ml) in 50% El Kolipher (Millipore-Sigma, St. Louis, MO) and 50% ethanol (Sigma-Aldrich) were diluted in sterile saline and administered intraperitoneally (i.p.) at a dose of 2 mg/kg [26] or intrathecally (i.t.) at a dose of 50 nM in 5 μl of phosphate-buffered saline (PBS) solutions. Control animals received an equivalent volume of the vehicle with proportional amounts of Cremophor EL and 95% dehydrated ethanol diluted in PBS. Signs of peripheral neuropathy with a similar phenotype to that in patients have been validated by multiple investigators in this non-tumor-bearing animal model, including an early and time-dependent development of mechanical and cold allodynia [25, 27, 28].

### Von Frey test for mechanical allodynia

Mechanical allodynia as a readout for CIPN was assessed as the hind paw withdrawal response to von Frey hair stimulation using the up-and-down method, as previously described [29]. Briefly, the mice were first acclimatized (1 h) in individual clear Plexiglas boxes on an elevated wire mesh platform to facilitate access to the plantar surface of the hind paws. Subsequently, a series of von Frey hairs (0.02, 0.07, 0.16, 0.4, 0.6, 1.0, and 1.4 g; Stoelting CO., Wood Dale, IL) were applied perpendicular to the plantar surface of hind paw. A test began with the application of the 0.6 g hair. A positive response was defined as a clear paw withdrawal or shaking. Whenever a positive response occurred, the next lower hair was applied, and whenever a negative response occurred, the next higher hair was applied. The testing consisted of six stimuli, and the pattern of response was converted to a 50% von Frey threshold, using the method described previously [30], by an investigator blinded to treatment until the end of the experiment.

### Cold plantar test for cold allodynia

The cold plantar assay was used to evaluate noxious cold [26, 31]. Briefly, animals were first placed individually into clear acrylic containers separated by black opaque dividers that were set on top of 3/160 borosilicate glass (Stemmerich Inc., St. Louis, MO) and allowed to acclimate for 20 min before testing. A dry ice pellet was applied to the hind paw through the glass and the time until hind paw withdrawal was recorded at 5-min intervals per mice, alternating paws, for a total of three trials, and the mean withdrawal latency was calculated by an investigator blinded to treatment until the end of the experiment.

### Drugs and intrathecal administration

For intrathecal injection, spinal puncture was made with a 30-gauge needle between the L5 and L6 level to deliver the reagents, as previously described [32–34]. We purchased the astrocyte inhibitor L-α-aminoadipate (L-α-AA) from Millipore-Sigma and administrated i.t. at a concentration of 50 nmol in 5 μl of PBS. Specific inhibitors PD98059 (Millipore-Sigma) targeting the upstream ERK kinase and SP600125 (Selleckchem, Houston, TX) targeting JNK kinase were used at doses reported in previous studies [22, 35]. Neutralizing antibodies for TNF-α (Catalog # AF-410) and SDF-1 (Catalog # MAB350) were purchased from R&D Systems (Minneapolis, MN) and administrated i.t. at 5 μg/site. Goat and mouse IgG (Santa Cruz Biotechnology, Dallas, TX) were respectively used at the same dose as controls.

### Astrocyte culture and intrathecal administration

To get high quality and large quantity of astrocytes, we prepared most astrocyte cultures from cerebral cortexes of post-neonatal mice (1–3 days old). The cerebral hemispheres were isolated and transferred to ice-cold Hank’s buffer, and the meninges were carefully removed. Tissues were then minced into about 1 mm pieces, triturated, filtered through 100- and then 40-μm strainers, and collected by centrifugation at 1000 g for 5 min. The cell pellets were broken with a pipette and resuspended in a medium containing 10% fetal bovine serum (FBS) in low-glucose DMEM (Thermo Fisher, Waltham, MA). After trituration, the cells were filtered through a 10-μm strainer, plated into six-well plates at a density of 2.5 × 10^5^ cells/cm^2^ or onto cover glasses for immunohistochemistry, and cultured for 10–12 days. The medium was replaced twice a week and once were grown to 95% confluence (about 10 days); 0.15 mM dibutyryl-cAMP (Millipore-Sigma) was added to induce differentiation. Three days later, the cells were used for immunohistochemistry, Western blot, and quantitative real-time RT-PCR or intrathecal injection experiments. Cultures were incubated with paclitaxel, recombinant TNF-α (R&D Systems), TLR4 inhibitor TLR4-IN-C34 (C34, Catalog # SML0832, Millipore-Sigma), or appropriate control solutions. For intrathecal injections, cultured astrocytes were harvested using 0.025% trypsin (Thermo Fisher) 1 h after vehicle or paclitaxel and washed thoroughly three times with PBS and centrifuged at 1000*g* for 5 min. About 2–4 × 10^4^ astrocytes in 10 μl of PBS were injected intrathecally between the L5 and L6 spinal levels with a 30-G needle to deliver the cells into the CSF, as previously described [36].

### Cell viability assay

Astrocyte culture were replated at same concentration (2.5 × 10^5^ cell/ml) and assessed for their viability 24 h after exposure to vehicle, paclitaxel, and TNF-α using the resazurin-based PrestoBlue® Cell Viability Reagent (Thermo Fisher). Briefly, 50 μl of PrestoBlue® reagent was added to culture media for 30 min and 200 μl of media collected for absorbance quantification. Absorbance was measured at 600 nm using an EnVision plate reader (PerkinElmer, Waltham, MA).

### Immunofluorescence

**Fig. 1:**
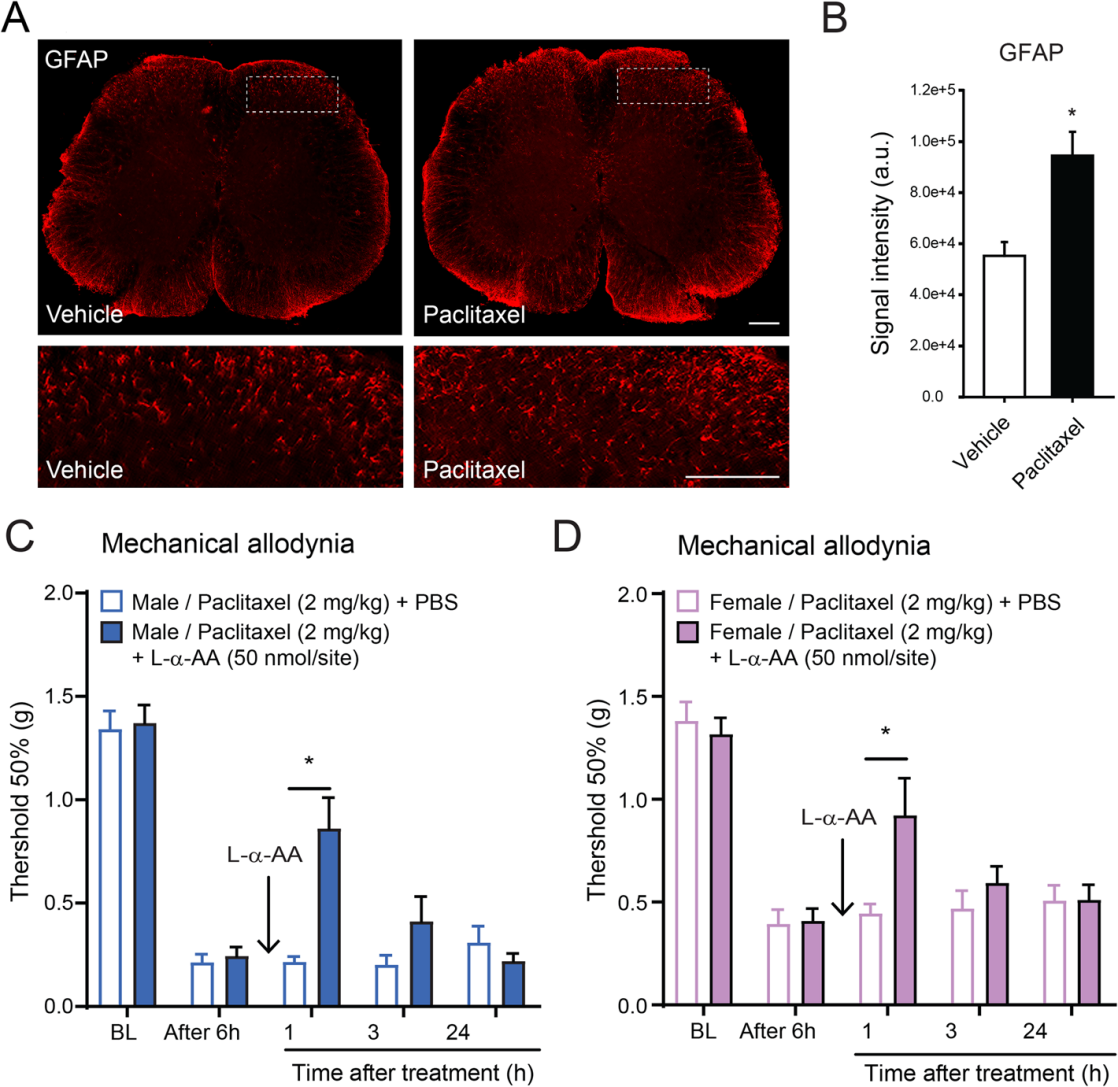
Systemic paclitaxel activates spinal astrocytes leading to mechanical allodynia. **a** Immunofluorescence showing GFAP expression in spinal cord sections of male mice 6 h after a single intraperitoneal (i.p.) injection of a vehicle control or paclitaxel in male mice. Dotted squares delineate quantification and magnified areas. Scale bar = 200 μm. **b** Quantification of immunofluorescence intensity of GFAP in the dorsal horn of the spinal cord, as delineated in **a** (*P < 0.05 compared to vehicle, *t* test, *n* = 5 per group). **c**, **d** Effect of intrathecal (i.t.) administration of the astrocytic inhibitor L-α-AA on mechanical allodynia induced by i.p. injection of paclitaxel in both male (**c**) and female (**d**) mice (*P < 0.05 compared to vehicle, ANOVA, *n* = 5 per group). BL baseline

Deeply anesthetized mice were perfused through the left ventricle of the heart with PBS, followed by 4% paraformaldehyde in PBS (PFA solution), and lumbar (L3–L5) spinal cord segment was removed and post-fixed in PFA solution overnight. Spinal cord tissues were transferred into 30% sucrose in PBS for 24 h, and then were sliced into 30-μm sections using a cryostat. For astrocyte cultures, cells were fixed with PFA solution for 20 min, washed with PBS, and processed for immunofluorescence. Spinal cord sections or astrocyte cultures were blocked for 1 h at room temperature with 1% BSA with 0.2% Triton X-100 in PBS (BSA solution) and incubated with glial fibrillary acidic protein primary antibody (GFAP, mouse, 1:500, Catalog # MAB360, Millipore-Sigma) overnight at 4 °C, followed by incubation with the secondary antibody anti-rabbit Alexa Fluor® 546 (1:1000, Thermo Fisher) for 1 h at room temperature. Images were captured under an Olympus BX63 fluorescent microscope using cellSens imaging acquisition software (Olympus, Center Valley, PA). A region of interest was drawn with cellSens within the dorsal horn including laminas I and II (Fig. 1a), and intensity quantifications of GFAP signal were performed comparing samples from all experimental groups, prepared with the same staining solutions, then measured using identical display parameters. Five to eight randomly selected spinal cord sections were used from each experimental animal, and background of a region outside of the tissue section and the area of the region of interest were used for normalization and quantification purposes, as previously described [18].

### Western blotting

Astrocyte culture was homogenized in a RIPA lysis buffer (Millipore-Sigma) complemented with a solution containing protease and phosphatase inhibitors (MS-SAFET, Millipore-Sigma). Protein samples were quantified using the Qubit® Protein Assay Kit (Thermo Fisher) according to the instruction of manufacturers, separated on SDS–PAGE gel (4–12% Bis-Tris Plus Gels, Thermo Fisher) and transferred to PVDF blots (Millipore-Sigma). Blots were blocked with Odyssey blocking buffer (TBS, LI-COR Biosciences, Lincoln, NE) and then probed with antibodies against p-JNK (rabbit, 1:500, Catalog # 9211, Cell Signaling, Danvers, MA) and glyceraldehyde 3-phosphate dehydrogenase (GAPDH, mouse, 1:10000, Catalog # ab8245, Abcam, Cambridge, MA) overnight. Blots were then washed and incubated with appropriate secondary fluorescent antibodies (LI-COR Biosciences) for 1 h at RT. Blot images were obtained by Odyssey CLx Imagine system (LI-COR Biosciences) and processed by Imagine Studio software (LI-COR Biosciences). The density of protein bands was analyzed with ImageJ software (National Institutes of Health, Bethesda, MD).

### Enzyme-linked immunosorbent assay (ELISA)

Protein samples were prepared in the same way as for Western blot analyses, and concentration determined by BCA Protein Assay (Pierce). Mouse TNF-α ELISA kit was purchased from R&D Systems, 50 μg of proteins was used, and ELISA was performed according to the protocol of the manufacturer. The standard curve was included in each experiment.

### Cytokine array

Protein samples were prepared in the same way as for Western blot analyses. We used the mouse Proteome ProfilerTM Arrays (Catalog # ARY006, R&D Systems) according to the protocol provided by the manufacturer, and cytokine expression levels were measured from four pooled samples. Samples were pooled to obtain the required amount and volume to cover the membranes and for a reliable use of the aforementioned arrays. Arrays were incubated with IRDye 800CW Streptavidin antibody (1:1000, Catalog # 926-32230, LI-COR Biosciences) and images Odyssey CLx Imagine system (LI-COR Biosciences) and processed by Imagine Studio software (LI-COR Biosciences). Protein densities were normalized by each individual array backgrounds and analyzed with ImageJ software (National Institutes of Health, Bethesda, MD).

### Quantitative real-time RT-PCR (qPCR)

Cultured astrocytes were lysed in TRIzol™ Reagent (Thermo Fisher), and total RNA was extracted using Direct-zol RNA MiniPrep kit (Zymo Research, Irvine, CA), which amount and quality were assessed by SimpliNano UV-Vis Spectrophotometer (General Electric, Boston, MA), and then converted into cDNA using a high-capacity cDNA reverse transcription kit (Thermo Fisher). Specific primers for GFAP, GAPDH, and various glutamate transporters as well as cytokines were obtained from PrimerBank [37]. Primer sequences are depicted in Additional file 3: Table S1. qPCR was performed on a QuantStudio™ 3 Real-Time PCR System (Thermo Fisher Scientific) using PowerUp SYBR™ Green Master Mix (Thermo Fisher Scientific). All samples were analyzed at least in duplicate and normalized by GAPDH expression, and the relative expression ratio per condition was calculated based on the method described by Pfaffl et al. [38, 39].

### Statistical analysis

Prism 8 software (Graphpad, La Jolla, CA) was used for statistical analysis. All data were expressed as mean ± SEM. Biochemical data were analyzed using Student’s *t* test or one-way analysis of variance (ANOVA) followed by Dunn’s post hoc test. Two-way repeated measured ANOVA was used to analyze multiple group data with multiple time points with Bonferroni post hoc test to determine on which days experimental groups differed. The criterion for statistical significance was set at *P* < 0.05.

## Results

### Paclitaxel activates spinal astrocytes leading to mechanical allodynia

To determine the activation of spinal astrocytes, we measured the protein levels of glial fibrillary acidic protein (GFAP) in spinal cord sections 6 h after systemic treatment of paclitaxel or vehicle solution (Fig. 1a). Immunofluorescence showed that paclitaxel induced a significant increase of GFAP in the superficial laminae of dorsal horn of the mouse spinal cord (area defined in Fig. 1a and quantified in Fig. 1b), which is consistent with findings in rat spinal cord sections reported in a previous study [18]. Although the expression of GFAP was only quantified in the superficial laminae of the spinal dorsal horn, a strong GFAP signal seems generalized to the whole spinal cord tissue including in areas surrounding the central canal and in the white matter of the spinal cord (Fig. 1a). Interestingly, these areas are intimately in contact with the cerebrospinal fluid (CSF), and although it is generally believed that paclitaxel does not cross the blood-brain barrier, low concentrations of paclitaxel can be detected in spinal cord tissue and CSF [24, 25]. Intrathecal injection into CSF of the astrocytic inhibitor L-α-aminoadipate (L-α-AA) is known to inhibit astrocyte activation and alleviate nerve injury- and inflammation-induced mechanical allodynia [23, 40]. We next examined whether mechanical allodynia elicited by paclitaxel would be inhibited by this intrathecal injection of the astrocytic inhibitor. We found that L-α-AA significantly reduced the development of mechanical allodynia evoked by systemic injection of paclitaxel in both male and female mice (Fig. 1c), similarly to a previous report for another chemotherapeutic agent [21].

### Intrathecal injection of paclitaxel elicits mechanical allodynia via astrocytes

**Fig. 2:**
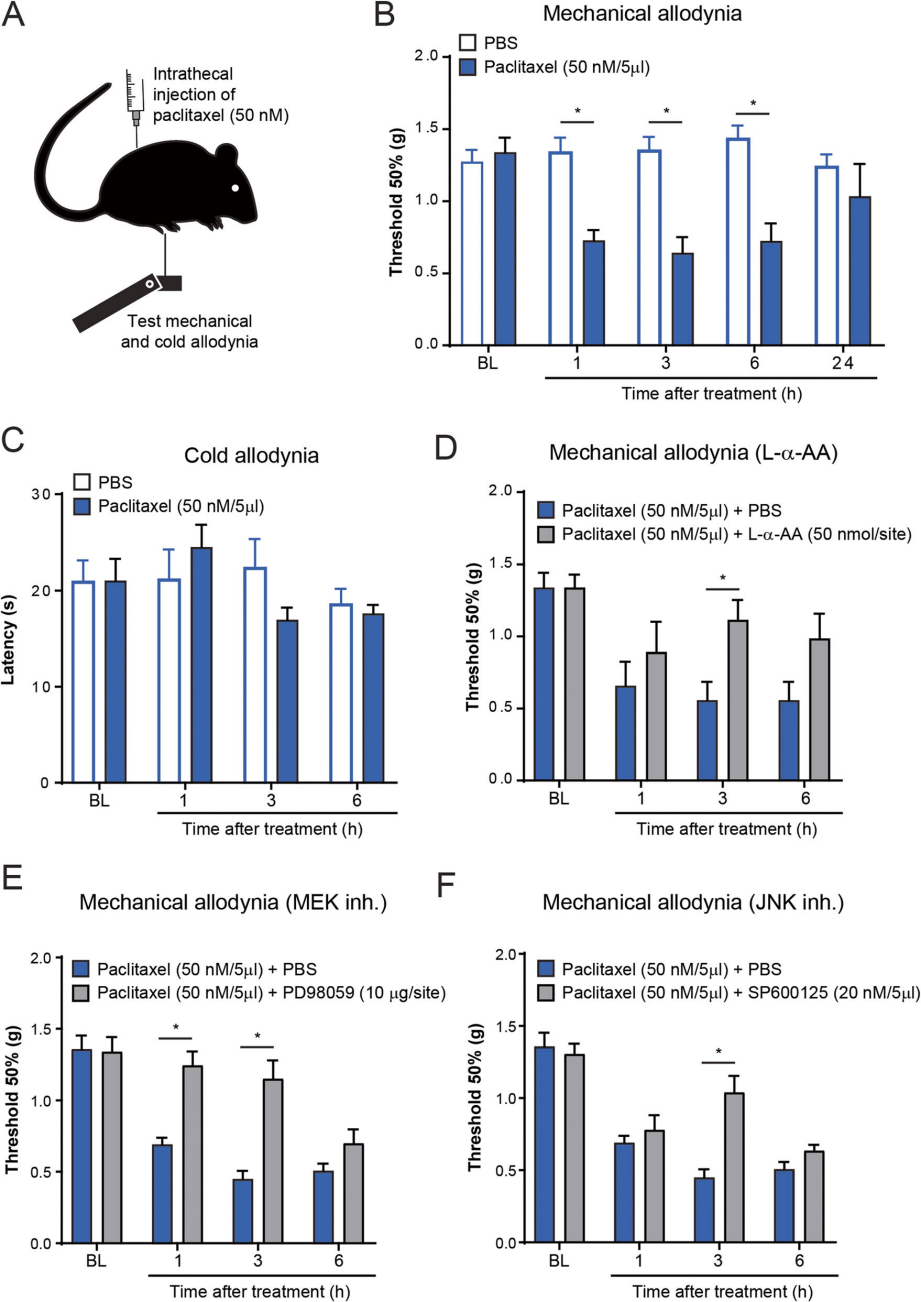
Intrathecal injection of paclitaxel elicits mechanical allodynia via astrocyte signaling. **a** Schematic illustration showing the experimental conditions and behavioral tests. **b**, **c** Effect of intrathecal injection of low amount paclitaxel on mechanical and cold allodynia in male mice (*P < 0.05 compared to vehicle, ANOVA, *n* = 5 per group). BL baseline. **d**–**f** Effect of intrathecal (i.t.) administration of the astrocytic inhibitor L-α-AA (**d**), as well as MEK (**e**) and JNK (**f**) inhibitors on mechanical allodynia induced by i.t. injection of paclitaxel in male mice (*P < 0.05 compared to PBS solution, ANOVA, *n* = 5 per group). BL baseline

The possibility of a direct spinal action of paclitaxel on astrocytes and mechanical allodynia was next tested by intrathecal injection of a low concentration of this chemotherapeutic agent (Fig. 2a). A concentration of 50 nM of paclitaxel was detected in spinal cord tissue [41] and increased mEPSC frequency in superficial dorsal horn neurons [25]. In line with this previous observation, we found that a single intrathecal injection of 50 nM of paclitaxel in 5 μl of PBS was sufficient to elicit a significant reduction in paw withdrawal threshold to mechanical stimuli, indicating the development of mechanical allodynia (Fig. 2b). This allodynia developed at 1 h and lasted for up to 6 h. In contrast, the same intrathecal injection of paclitaxel showed no effect in paw withdrawal threshold to cold stimuli, indicating the absence of a cold allodynia (Fig. 2c). Notably, intrathecal injection of a vehicle control had no effect on both paw withdrawal thresholds (Fig. 2b, c). Similar to our previous result using the astrocytic inhibitor L-α-AA, we found that this inhibitor also significantly reduced the development of mechanical allodynia evoked by intrathecal injection of paclitaxel (Fig. 2d). Various mitogen-activated protein kinase (MAPK) family members are activated in spinal glial cells after nerve injury [14]. In particular, ERK is activated sequentially in microglia and astrocytes, and JNK is activated persistently in astrocytes [23, 35]. Intrathecal injection of the ERK kinase signaling inhibitor (10 μg/site) or JNK inhibitor SP600125 (20 nM/5 μl) both significantly reversed the paclitaxel-induced mechanical allodynia (Fig. 2e, f). These findings suggest that low concentrations of paclitaxel are sufficient to induce mechanical allodynia partially via astrocytic signaling.

### Paclitaxel induces the activation of cultured astrocytes independently from TLR4 signaling

**Fig. 3:**
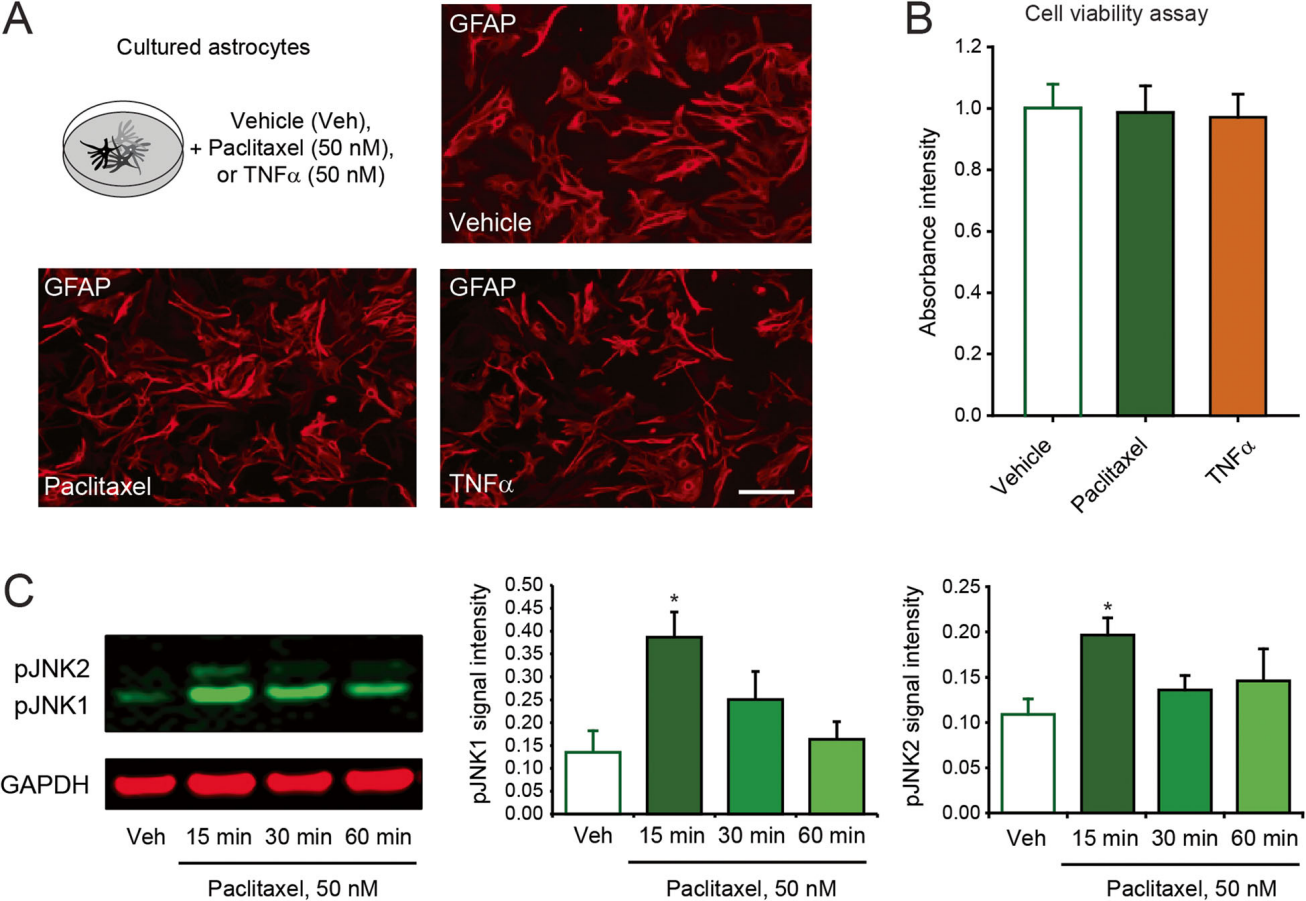
Paclitaxel induces the activation of cultured astrocytes. **a** Schematic illustration of the experimental conditions and immunofluorescence of GFAP in cultured astrocytes 24 h after exposure to vehicle control, paclitaxel, or TNF-α. Scale bar = 20 μm. **b** Quantification of astrocyte viability 24 h after incubation with vehicle control, paclitaxel, or TNF-α. **c** Western blot showing the phosphorylation/activation of JNK in cultured astrocytes after paclitaxel, as well as density of pJNK1 and pJNK2 bands, which are normalized to and expressed as ratio of the GAPDH loading control (*P < 0.05 compared to vehicle, ANOVA, *n* = 4 per group)

To determine whether paclitaxel can directly activate astrocytes, we stimulated primary culture of astrocytes with 50 nM of paclitaxel or TNF-α (Fig. 3a). Because paclitaxel is known as a cellular toxin, we first assessed the quality of our cultured astrocytes (Fig. 3a) and demonstrated a comparable viability among vehicle-, paclitaxel-, and TNFα-treated cultures (Fig. 3b). We then tested the phosphorylation of JNK, which has been previously involved in the activation of astrocytes by TNF-α and in the development of neuropathic pain [22, 42]. The expression levels of two main isoforms of pJNK (pJNK1 and pJNK2) were very low or absent in vehicle-treated cultures. However, paclitaxel treatment induced a rapid, although transient, activation (phosphorylation) of JNK1 and JNK2 (Fig. 3c). This phosphorylation appeared to be more prominent for JNK1 and reached to a peak within 15 min, similarly to what was observed for TNF-α [22]. Although paclitaxel has been suggested to engage TLR4 in microglia and neurons [16, 25], we found that activation of JNK was intact when the cultures were treated with the TLR4 inhibitor C34 [43] during the paclitaxel stimulation (Additional file 1: Figure S1). This is consistent with the report of the absence of TLR4 in cultured astrocytes [44]. Together, these results suggest that paclitaxel can activate astrocytes via the phosphorylation of JNK, but they probably engage different and TLR4 independent signaling pathways than neurons.

### Paclitaxel and TNF-α induce different cytokines in cultured astrocytes

**Fig. 4:**
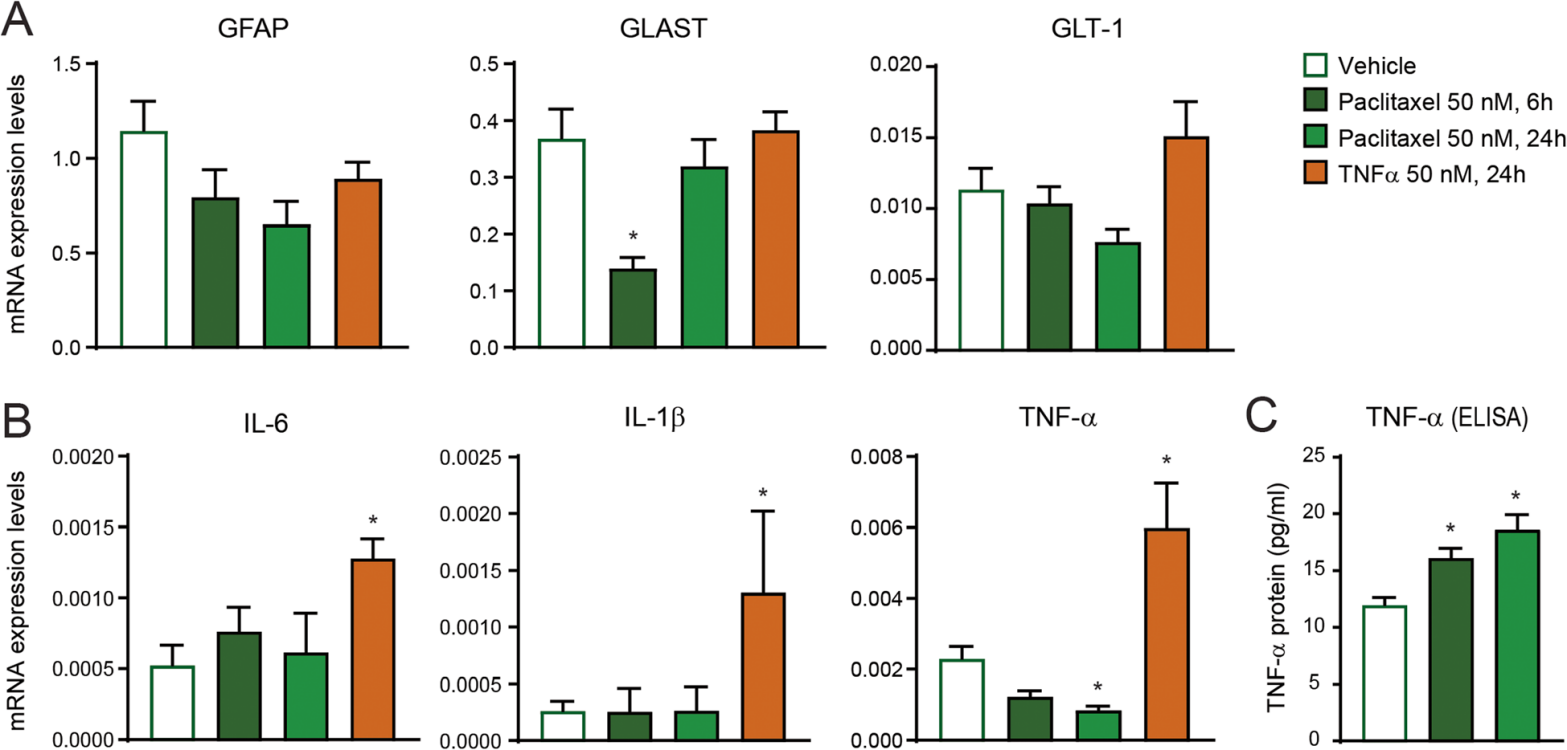
Paclitaxel and TNF-α induce different expression of cytokines in cultured astrocytes. **a**, **b** Quantitative transcriptional analyses of GFAP, glutamate transporters (GLAST and GLT-1), and cytokines (TNF-α, IL-6, IL-1β) and chemokines in cultured astrocytes after exposure to vehicle control (24 h), paclitaxel (6 h and 24 h), or TNF-α (24 h) (*P < 0.05 compared to vehicle, *t* test, *n* = 4–5 per group). **c** TNF-α expression, revealed by ELISA analysis, in primary cultures of astrocytes after exposure to paclitaxel (6 h and 24 h) (*P < 0.05 compared to vehicle, *t* test, *n* = 4 per group)

Microtubule stabilizers such as paclitaxel can change directly the expression and distribution of GFAP and indirectly regulate the astrocytic glutamate transporters that may be responsible for increases in synaptic transmission and paclitaxel-induced neuropathic pain [18, 45]. However, no or minimal transcriptional changes were observed in cultured astrocytes after paclitaxel and TNF-α exposure (50 nM for 6 h or 24 h) for GFAP, as well as GLAST and GLT-1 (Fig. 4a), two glutamate transporters that are implicated in neuropathic pain and are expressed predominantly in astrocytes [18, 46]. Interestingly, GLAST is temporary downregulated at 6 h partially mimicking previous findings in vivo [18, 45]. Activation of JNK in astrocytes by TNF-α plays an important role in the expression of pro-inflammatory cytokines contributing to neuropathic pain [22, 32]. Although transcriptional analyses of cultured astrocytes showed no changes in pro-inflammatory cytokines such as interleukin (IL)-1β and IL-6, a significant decreased for TNF-α was observed after paclitaxel exposure (Fig. 4b). To note, all these pro-inflammatory cytokines were increased in cultured astrocytes after TNF-α exposure (Fig. 4b). The significant decrease of TNF-α after paclitaxel prompted us to ask whether post-transcriptional changes would regulate cytokines and chemokines after exposure to paclitaxel. We found by ELISA that the protein levels of TNF-α were significantly increased after paclitaxel, suggesting indeed compensatory post-transcriptional changes (Fig. 4c). We also performed a preliminary screening using a cytokine array (blot) that contains 40 different cytokine and chemokine antibodies (Additional file 2: Figure S2A). We covered the blots with cell lysates from cultured astrocytes incubated with vehicle control or paclitaxel (Additional file 2: Figure S2B). In particular, incubation with paclitaxel induced modest but significant changes in TNF-α (1.16-fold, *P* < 0.01) and stromal cell-derived factor 1 (SDF-1, alias CXCL12, 1.07-fold, *P* < 0.001) (Additional file 2: Figure S2C and Additional file 4: Table S2). The results suggest that brief exposure to paclitaxel regulates the post-transcriptional production of different cytokines in astrocytes. After this initial screening, we chose to further characterize TNF-α and SDF-1 since they are strongly implicated in astrocyte activation [47] in animal models of inflammatory and neuropathic pain [48–50].

### Intrathecal injection of paclitaxel-activated astrocytes elicit allodynia via TNF-α and SDF-1

**Fig. 5:**
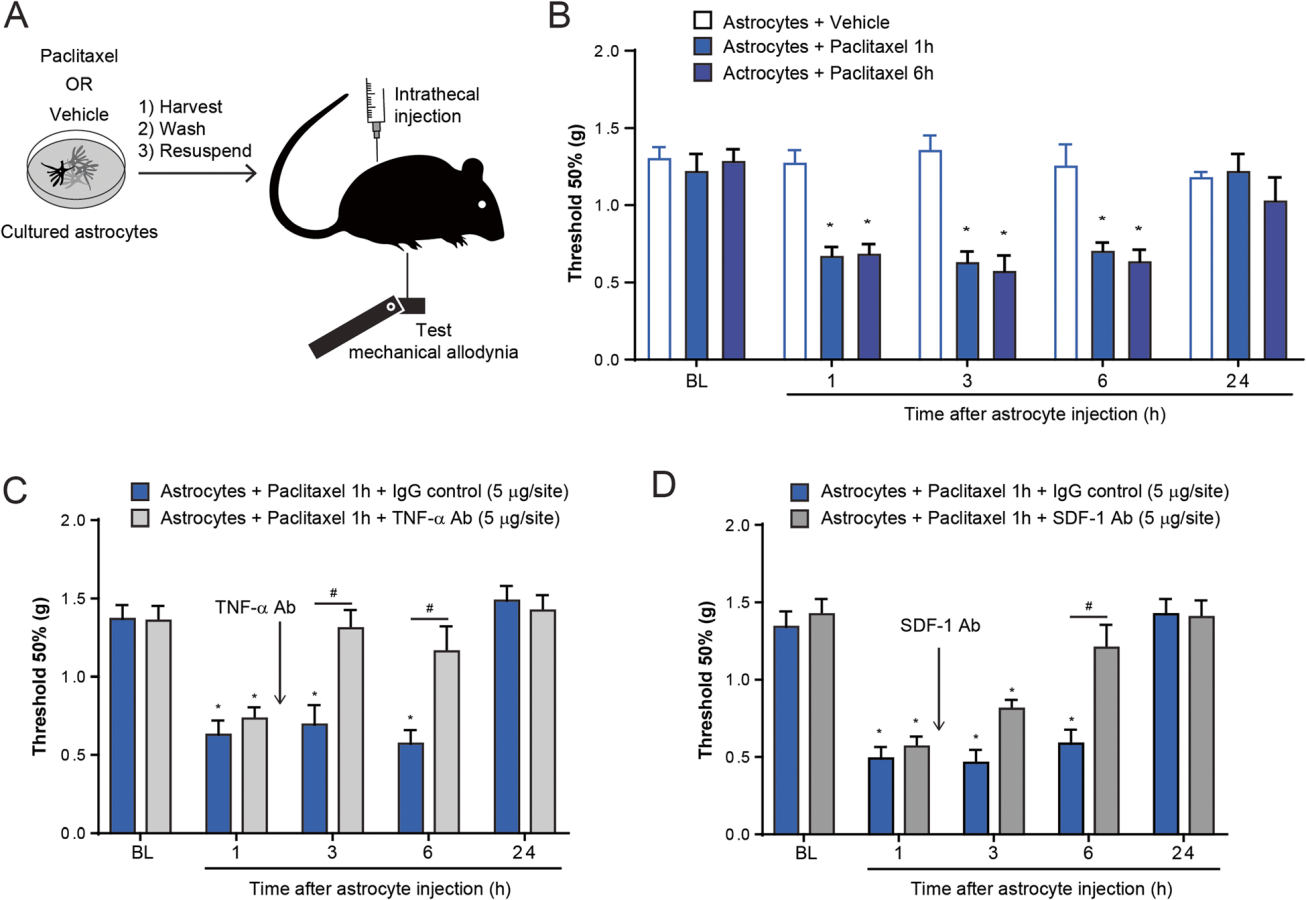
Intrathecal injection of paclitaxel-activated astrocytes elicit allodynia via TNF-α and SDF-1. **a** Schematic illustration showing the experimental conditions of cultured astrocytes, intrathecal injection, and behavioral tests. **b** Effect of intrathecal (i.t.) injection of paclitaxel-activated astrocytes (astrocytes cultured with vehicle, paclitaxel for 1 or 6 h) on mechanical allodynia in male mice (*P < 0.05 compared to vehicle, ANOVA, *n* = 5 per group). **c**, **d** Effect of intrathecal (i.t.) administration of TNF-α or SDF-1 neutralizing antibody on mechanical allodynia induced by i.t. injection of paclitaxel-activated astrocytes in male mice (astrocytes cultured with paclitaxel for 1 h, *P < 0.05 compared to IgG control, ANOVA, *n* = 5 per group)

To determine whether paclitaxel-activated astrocytes are sufficient to induced pain sensitization, we prepared cultured astrocytes, which were then stimulated with a vehicle control or paclitaxel (50 nM for 1 h or 6 h). After harvesting these astrocytes, we washed them thoroughly three times with PBS to remove the paclitaxel and collected the astrocytes for intrathecal injection in naïve mice (Fig. 5a). We found a dramatic reduction in paw withdrawal threshold after intrathecal injection of paclitaxel-stimulated astrocytes, indicating the development of mechanical allodynia (Fig. 5b). This allodynia developed at 1 h and lasted for up to 6 h. Notably, mice that received intrathecal injection of vehicle-stimulated did not develop mechanical allodynia (Fig. 5b). To test the hypothesis that paclitaxel-activated astrocytes release TNF-α and SDF-1 to generate tactile allodynia in naïve animals, we intrathecally injected a TNF-α or SDF-1 neutralizing antibody at 1 h after intrathecal injection of paclitaxel-activated astrocytes. At a dose (5 μg/site) that is effective in reducing glia-driven pain hypersensitivity [33], the TNF-α neutralizing antibody completely reversed mechanical allodynia induced by paclitaxel-activated astrocytes (Fig. 5c). Similarly, SDF-1 neutralizing antibody (5 μg/site) also reversed mechanical allodynia induced by paclitaxel-activated astrocytes (Fig. 5d). In contrast, intrathecal injection of the control immunoglobulin G (IgG) had no effect on mechanical allodynia (Fig. 5c, d). Collectively, these results suggest that paclitaxel-activated astrocytes are sufficient to induce mechanical allodynia in naïve mice, which is caused by the release of TNF-α and SDF-1.

## Discussion

Paclitaxel is associated with acute pain syndrome for which the underlying mechanisms are poorly understood hampering the development of new and much needed therapeutic treatments. Here, we uncovered the role of spinal astrocytes for the development of mechanical allodynia in a preclinical model of P-APS and showed that intrathecal injections of low levels of paclitaxel or paclitaxel-activated astrocytes are sufficient to produce mechanical allodynia.

Activation of spinal microglia and astrocytes is emerging as key mechanism underlying chronic pain [15]. Activation of astrocytes has been shown to contribute to the development of chronic pain in various preclinical conditions including in animal models of chemotherapy-induced neuropathic pain. This activation is generally slow to develop and preceded by microglial reaction, which is known to drive astrocyte activation [14]. Although there is evidence supporting a role of microglia in chemotherapy-induced pain [16, 51, 52], astrocytes can be rapidly activated in the apparent absence of microglia reaction after exposure to the chemotherapeutic agents paclitaxel, oxaliplatin, and bortezomib [18, 53]. Our results are in line with these observations and show that activation of astrocytes, which we assessed by upregulation of GFAP, occurs in few hours after a single systemic injection of paclitaxel. Furthermore, we also demonstrated that the mechanical allodynia associated with this activation is reversed by the astrocytic inhibitor L-α-AA in both male and female mice. This result is in line with our previous study indicating sex-dependent microglial but not astrocytic signaling in the spinal cord in nerve injury-induced neuropathic pain [54]. The rapid upregulation of GFAP and analgesic effect of astrocytic inhibitors rise the question about how astrocyte activation occurs after delivery of paclitaxel.

Several studies have demonstrated that low levels (~ 5–80 nM) of paclitaxel penetrate into the CSF and are detectable in the spinal cord in the initial hours and days of treatments in animals and patients [24, 25, 55, 56]. Here, we show an upregulation of GFAP signal in the dorsal horn of the spinal cord, but we have also observed a strong signal in the areas around the central canal and in white matter. How astrocytes in these areas can participate to pain is still unclear, but extracellular mediators such cytokines can diffuse through the spinal cord and CSF and mediate pain processing. For instance, intrathecal administration of TNF-α elicits pain [22, 57] or cervical increase of TNF-α induces mechanical allodynia in rodent hind paws [58]. Interestingly, the strong GFAP signal in these areas, which are highly exposed to the CSF, may indicate that amounts of paclitaxel which penetrate into the CSF can be sufficient to directly activate astrocytes and thus elicit P-APS. It has been reported that paclitaxel in CSF and spinal cord tissue can direct the development and progress of P-APS [56]. Here, we used a concentration of 50 nM of paclitaxel that was previously reported in the CSF patients after a single intravenous injection of paclitaxel [59] and acute application shown to increased synaptic transmission in rat spinal cord slices [25]. Using this concentration, we show that intrathecal injection into the intrathecal space of a small amount of paclitaxel is sufficient to induce mechanical allodynia, which is reversed by the astrocytic inhibitor L-α-AA. Previous study showed that phosphorylation of ERK and, in particular, JNK in spinal astrocytes plays an important role in neuropathic pain [14]. Both inhibition of ERK and JNK reduced this paclitaxel-induced mechanical allodynia, suggesting a major participation of astrocytic signaling to P-APS. We and other groups have reported the development of cold allodynia in mice receiving systemic injections of paclitaxel [26, 60, 61]. Interestingly, our intrathecal injection of paclitaxel failed to replicate this particular allodynia. Recently, it has been suggested that peripheral sensory neurons are essential for cold allodynia, whereas mechanical allodynia is mostly dependent on the activation of the immune system after peripheral nerve injury [62]. Although it is tempting to conclude from this study and our behavioral results that our intrathecal injection of paclitaxel mostly targets and activates non-neuronal cells such as astrocytes, we cannot exclude that this intrathecal drug administration may also impact the activity of other cells in the spinal cord and dorsal root ganglia. It is therefore possible that the aforementioned behaviors can be only partially driven by astrocytes.

To determine the direct and exclusive role of paclitaxel in astrocytes, we prepared primary cultures of astrocytes and mimic the in vivo conditions of astrocytes with brief exposure to low amount of paclitaxel. It has been demonstrated that p-JNK is increased in spinal astrocytes in chronic pain conditions, including after paclitaxel injections [20]. This increase is induced in vivo and in vitro by diverse stress-related stimuli and pro-inflammatory cytokines such as TNF-α [22, 23]. Although paclitaxel induces no major changes in the morphology and viability of cultured astrocytes, it significantly increased the expression of p-JNK in minutes as previously reported in astrocytes stimulated with TNF-α [22]. In line with the predominant expression of JNK1 in astrocytes [22], we observed a stronger increase in the expression of this isoform after paclitaxel. Although paclitaxel has been suggested to engage TLR4 in microglia and neurons [16, 25], cultured astrocytes have been reported to lack the expression of TLR4 [44] and we found that activation of JNK was independent from TLR4. Further studies are needed to investigate how paclitaxel interacts with astrocytes. However, it has been reported that paclitaxel can change intermediate filaments including GFAP in various cells and can induce the activation of JNK pathway leading to the apoptosis of carcinoma cells [45, 63]. The exact upstream mechanisms by which paclitaxel engages GFAP and JNK remain unclear and require further studies.

In contrast, JNK downstream signaling is well-known for engaging several transcriptional factors such as c-Jun and induces gene transcription in astrocytes [23]. However, no dramatic changes were observed by our transcriptional analysis of paclitaxel-treated astrocytes. We, as shown here, and others have observed that GFAP levels are increased in astrocytes of mice injected with paclitaxel [18, 20], and it has been reported that paclitaxel induces early downregulation of multiple glutamate transporters [18]. However, we found that only GLAST mRNA is temporarily decreased in cultured astrocytes 6 h after paclitaxel. It is therefore possible that these transporters are posttranscriptionally regulated or in vivo astrocytes received additional inputs from other cells causing additional changes. This is a clear limitation of our study, and it should be further investigated in follow-up studies. It is also surprising that TNF-α- and paclitaxel-treated astrocytes present similar pJNK activation [22], but robust transcriptional increases in pro-inflammatory cytokines are only observed in TNF-α-treated astrocytes. However, these results are in line with a previous report showing no transcriptional changes for IL-1β, IL-6, and TNF-α in the spinal cord tissue after paclitaxel [18]. Changes in pro-inflammatory cytokines can occur at translational levels. Indeed, recent studies have reported increased TNF-α and IL-1β in the spinal cord tissues and astrocytes in mice treated with various chemotherapeutic agents, including paclitaxel [19, 21, 64].

Our data shows that TNF-α and SDF-1 proteins are seemingly increased in and released by paclitaxel-activated astrocytes into CSF and spinal cord tissue. TNF-α is an essential trigger of inflammatory cascades that underline the pathogenesis of pain. TNF-α can directly and rapidly enhance excitatory synaptic transmission in dorsal horn neurons [65] and can also further activates glial cells [22, 66]. Interestingly, spinal administration of TNF-α is sufficient to induce mechanical allodynia [22, 57], and systemic injection of TNF-α inhibitor etanercept significantly attenuated paclitaxel-established mechanical and cold allodynia by putative actions on both peripheral and central TNF-α signaling [17, 67]. Similarly, blockade of SDF-1 signaling ameliorated the paclitaxel-induced mechanical allodynia [50]. Consistently, SDF-1 is produced by glial cells and neurons in the central nervous system [68], and epigenetic upregulation of SDF-1 expression has been demonstrated in mice receiving paclitaxel injections [50]. Of note, it has been reported that TNF-α promotes the activation of NF-κB by SDF-1 [47] that can sustain the production of pro-inflammatory cytokines underlying the development and progression of paclitaxel-induced neuropathic pain [19, 21, 64, 69].

Paclitaxel has been suggested to elicit pain by engaging microglia and neurons via TLR4 [16, 25]. However, we have showed that the astrocytic inhibitor L-α-AA significantly attenuated P-APS and found that paclitaxel-induced activation of astrocytes was independent from TLR4. Furthermore, recent evidence suggests that activation of astrocytes may be sufficient to produce pain. We have previously reported that spinal injection of TNF-α-activated astrocytes produces a robust mechanical allodynia by the release of MCP-1 [32]. Here, we provide direct evidence that intrathecal injection of paclitaxel-activated astrocytes is sufficient to induce mechanical allodynia compared to injection of control astrocytes. Furthermore, we demonstrate the functional role of the release of pro-inflammatory cytokines from paclitaxel-activated astrocytes since the mechanical allodynia induced by these astrocytes is reversed by both TNF-α and SDF-1 neutralizing antibodies. It remains to be investigated whether paclitaxel-activated astrocytes may also activate other spinal cells (e.g., microglia or oligodendrocytes) to increase the release of the pro-inflammatory cytokines and thus actively participate to induce this mechanical allodynia. Perhaps, it is also important that there is an apparent delay in the analgesic action of the SDF-1 antibody compared to the TNF-α neutralizing antibody, raising the possibility that TNF-α release precedes and controls SDF-1 production [47] and thus may represent a better therapeutic target for the treatment of paclitaxel-associated pain.

## Conclusion

In the present study, we have demonstrated that astrocytes are involved in the development of paclitaxel-associated acute pain syndrome. We have also established that paclitaxel directly activates astrocytes, and paclitaxel-activated astrocytes are sufficient to induce mechanical allodynia via TNF-α and SDF-1. Future studies should be conducted to investigate whether other chemotherapeutic agents or multiple injections of paclitaxel recapitulate and further activate astrocytes or other cells by engaging similar signaling. However, it is now clear that paclitaxel not only accumulates in dorsal root ganglia [70] but also penetrates the CSF and spinal cord tissue where it can directly activate astrocytes and cause pain. Therefore, future therapeutic approaches aiming to reduce paclitaxel-associated pain might be made more effective by targeting both peripheral and central pathological changes.

## Supplementary information

**Additional file 1: Figure S1.** Paclitaxel induces the activation of JNK in cultured astrocytes independently from TLR4 signaling. Western blot showing the phosphorylation/activation of JNK in cultured astrocytes 15 min after paclitaxel, as well as density of pJNK1, pJNK2 bands, which are normalized to and expressed as ratio of the GAPDH loading control (C34 = TLR4 inhibitor TLR4-IN-C34, ANOVA, *n* = 4 per group).

**Additional file 2: Figure S2.** Cytokine array indicates potential regulation of chemokines in astrocytes after paclitaxel exposure. A, Illustration of the cytokine array with 40 cytokines in duplicates (PC = positive control). B and C, Blot arrays and volcano plot reveal the protein regulation of various cytokines such TNF-α and SDF-1 24 h after exposure to paclitaxel (*P* < 0.05 compared to vehicle, t-test, *n* = 2 assays, 4 samples were pooled together see methods).

**Additional file 3:** **Table S1.** qPCR primer sequences.

**Additional file 4:** **Table S2.** Volcano plot data.

## Data Availability

Please contact author for raw data requests.

## Abbreviations

CSF: Cerebrospinal fluid
ERK: Extracellular signal-regulated kinase
GFAP: Glial fibrillary acidic protein
JNK: c-Jun N-terminal kinase
L-α-AA: L-α-aminoadipate
MCP-1: Monocyte chemoattractant protein-1
P-ACP: Paclitaxel-associated acute pain syndrome
SDF-1: Stromal cell-derived factor 1
TNF-α: Tumor necrosis factor alpha
