# Supplementary figures and images for: Paclitaxel-activated astrocytes produce mechanical allodynia in mice by releasing tumor necrosis factor-α and stromal-derived cell factor 1
Source: J Neuroinflammation. 2019 Nov 10;16:209. doi: 10.1186/s12974-019-1619-9 (PMC6842526; doi:10.1186/s12974-019-1619-9)

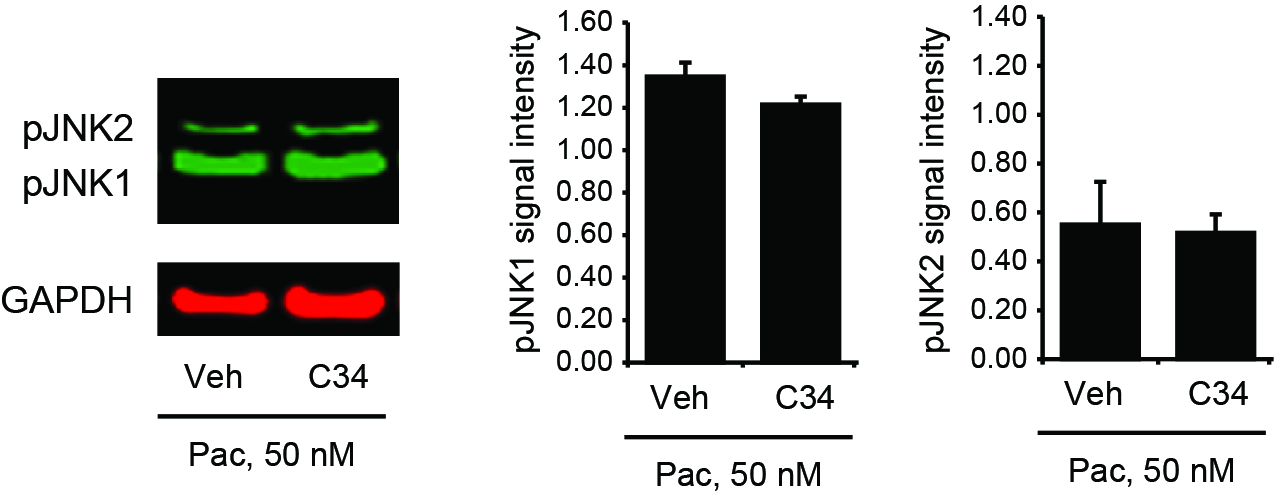

Supplement: Supplementary file 1 — Additional file 1: Figure S1. Paclitaxel induces the activation of JNK in cultured astrocytes independently from TLR4 signaling. Western blot showing the phosphorylation/activation of JNK in cultured astrocytes 15 min after paclitaxel, as well as density of pJNK1, pJNK2 bands, which are normalized to and expressed as ratio of the GAPDH loading control (C34 = TLR4 inhibitor TLR4-IN-C34, ANOVA, n = 4 per group). [file 12974_2019_1619_MOESM1_ESM.tif]

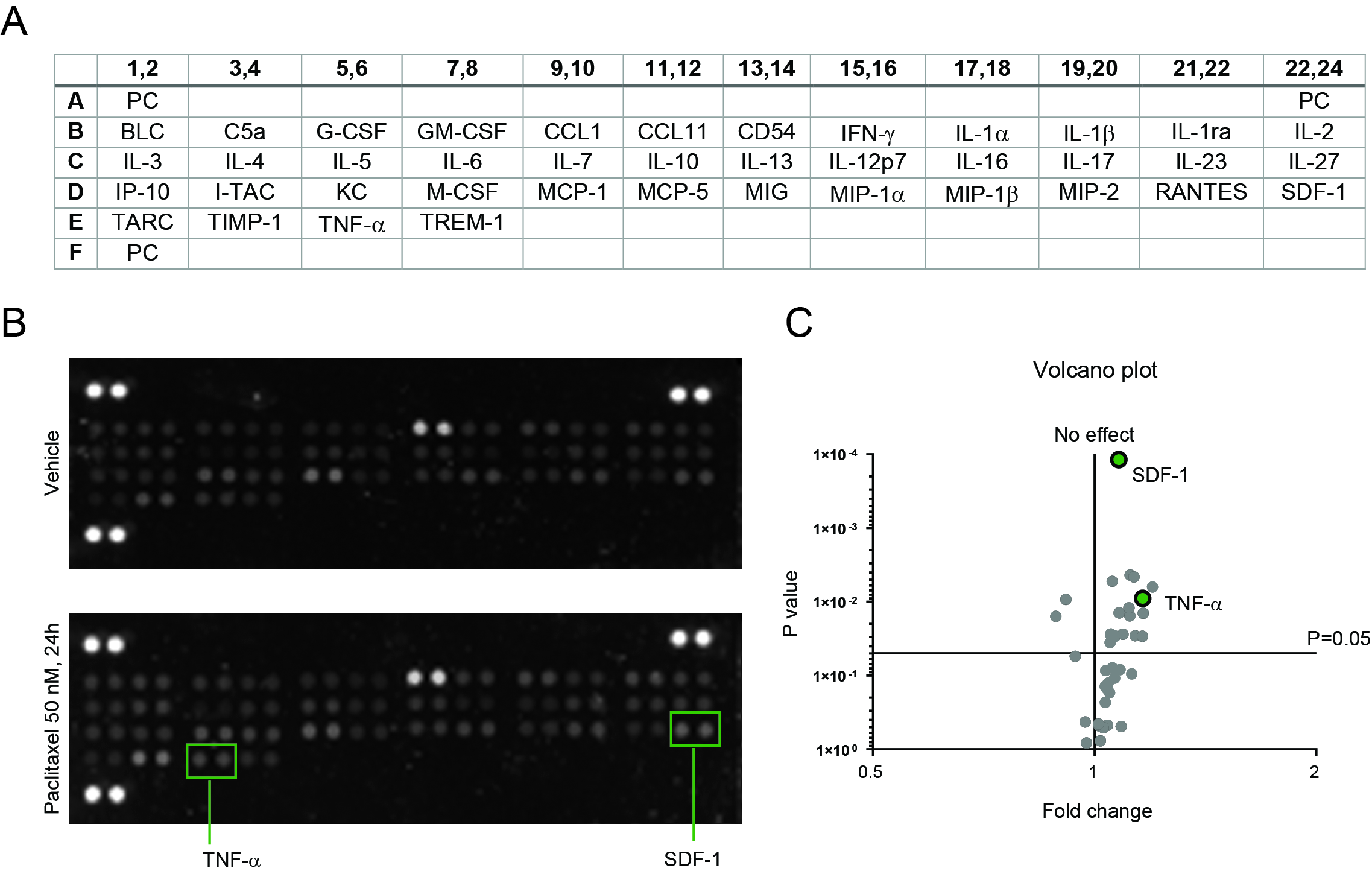

Supplement: Supplementary file 2 — Additional file 2: Figure S2. Cytokine array indicates potential regulation of chemokines in astrocytes after paclitaxel exposure. A, Illustration of the cytokine array with 40 cytokines in duplicates (PC = positive control). B and C, Blot arrays and volcano plot reveal the protein regulation of various cytokines such TNF-α and SDF-1 24 h after exposure to paclitaxel (P < 0.05 compared to vehicle, t-test, n = 2 assays, 4 samples were pooled together see methods). [file 12974_2019_1619_MOESM2_ESM.tif]
