# Supplementary material for: Paclitaxel-activated astrocytes produce mechanical allodynia in mice by releasing tumor necrosis factor-α and stromal-derived cell factor 1
Source: J Neuroinflammation. 2019 Nov 10;16:209. doi: 10.1186/s12974-019-1619-9 (PMC6842526; doi:10.1186/s12974-019-1619-9)
Supplement: Supplementary file 3 — Additional file 3: Table S1. qPCR primer sequences. [file 12974_2019_1619_MOESM3_ESM.doc]

**Additional file 3: Table S1.** qPCR primer sequences.

| **Gene** | **Forward primer** | **Reverse Primer** | **Accession #** |
| --- | --- | --- | --- |
| **GFAP** | CGGAGACGCATCACCTCTG | AGGGAGTGGAGGAGTCATTCG | NM_001131020.1 |
| **GLAST** | ACCAAAAGCAACGGAGAAGAG | GGCATTCCGAAACAGGTAACTC | NM_148938.3 |
| **GLT-1** | ACAATATGCCCAAGCAGGTAGA | CTTTGGCTCATCGGAGCTGA | NM_011393.2 |
| **TNFα** | TCTCATGCACCACCATCAAGGACT | TGACCACTCTCCCTTTGCAGAACT | NM_013693.3 |
| **IL-6** | ACAACCACGGCCTTCCCTAC | TCCACGATTTCCCAGAGAACA | NM_001314054.1 |
| **IL-1β** | CAACCAACAAGTGATATTCTCCATG | GATCCACACTCTCCAGCTGCA | NM_008361.4 |
| **MCP-1** | TTAAAAACCTGGATCGGAACCAA | GCATTAGCTTCAGATTTACGGGT | NM_011333.3 |
| **GAPDH** | AGGTCGGTGTGAACGGATTTG | GGGGTCGTTGATGGCAACA | NM_001289726.1 |
