# Supplementary material for: Paclitaxel-activated astrocytes produce mechanical allodynia in mice by releasing tumor necrosis factor-α and stromal-derived cell factor 1
Source: J Neuroinflammation. 2019 Nov 10;16:209. doi: 10.1186/s12974-019-1619-9 (PMC6842526; doi:10.1186/s12974-019-1619-9)
Supplement: Supplementary file 4 — Additional file 4: Table S2. Volcano plot data. [file 12974_2019_1619_MOESM4_ESM.doc]

**Additional file 4: Table 3.** Volcano plot data.

| **Cytokine ID** | **Fold change** | **P value** |
| --- | --- | --- |
| | | BLC | | --- | | C5a | | G-CFS | | GM-CSF | | CCL1 | | CCL11 | | CD54 | | IFNy | | IL-1a | | IL-1b | | IL-1ra | | IL-2 | | IL-3 | | IL-4 | | IL-5 | | IL-6 | | IL-7 | | IL-10 | | IL-13 | | IL-12p70 | | IL-16 | | IL-17 | | IL-23 | | IL-27 | | IP-10 | | I-TAC | | KC | | M-CSF | | MCP-1 | | MCP-5 | | MIG | | MIP-1a | | MIP-1b | | MIP-2 | | RANTES | | SDF-1 | | TARC | | TIMP-1 | | TNFa | | TREM-1 | | | --- | --- | --- | --- | --- | --- | --- | --- | --- | --- | --- | --- | --- | --- | --- | --- | --- | --- | --- | --- | --- | --- | --- | --- | --- | --- | --- | --- | --- | --- | --- | --- | --- | --- | --- | --- | --- | --- | --- | --- | --- | | | 1.041411 | | --- | | 1.117428 | | 1.067112 | | 1.092643 | | 1.041393 | | 1.018402 | | 1.034481 | | 1.050129 | | 1.05224 | | 1.013731 | | 1.123135 | | 1.116175 | | 0.975331 | | 1.164266 | | 1.131536 | | 1.135663 | | 1.05692 | | 0.971215 | | 1.035515 | | 1.034037 | | 1.064711 | | 1.087256 | | 1.043463 | | 1.051618 | | 1.082389 | | 1.113638 | | 0.941408 | | 1.080358 | | 0.886034 | | 1.057446 | | 1.040555 | | 0.914182 | | 1.047962 | | 1.027714 | | 1.012271 | | 1.074482 | | 1.161618 | | 1.198196 | | 1.162537 | | 1.059581 | | | 0.472584 | | --- | | 0.004335 | | 0.029122 | | 0.027484 | | 0.152787 | | 0.767345 | | 0.231096 | | 0.03566 | | 0.028274 | | 0.478444 | | 0.094565 | | 0.015425 | | 0.818113 | | 0.014236 | | 0.004574 | | 0.028824 | | 0.005277 | | 0.426394 | | 0.0854 | | 0.140018 | | 0.108232 | | 0.488571 | | 0.126361 | | 0.027114 | | 0.08291 | | 0.012042 | | 0.054853 | | 0.0141 | | 0.015736 | | 0.078331 | | 0.152059 | | 0.00923 | | 0.169893 | | 0.509227 | | 0.453223 | | 0.000113 | | 0.02928 | | 0.006281 | | 0.008928 | | 0.030082 | |
